# The paradox of neutral carbonate budgets on coral-dominated reefs

**DOI:** 10.1098/rsos.260604

**Published:** 2026-10-21

**Authors:** Elizabeth Cabrera-Rivera, Didier de Bakker, Ana L. Molina-Hernández, Francisco Medellín-Maldonado, Rodolfo Rioja-Nieto, Alexis E. Medina Valmaseda, Esmeralda Pérez-Cervantes, Chris T. Perry, Lorenzo Álvarez-Filip

**Affiliations:** ^1^ Biodiversity and Reef Conservation Laboratory, Unidad Académica de Sistemas Arrecifales, Instituto de Ciencias del Mar y Limnología, Universidad Nacional Autónoma de México, Puerto Morelos, México; ^2^ Geography, Faculty of Environment, Science and Economy, University of Exeter, Exeter, UK; ^3^ Programa de Investigación Espacial en Ambientes Costeros y Marinos, Facultad de Ciencias, Universidad Nacional Autónoma de México, UMDI-Sisal, Sisal, Yucatán, México; ^4^ Instituto de Investigaciones Oceanológicas, Universidad Autónoma de Baja California, Ensenada, México; ^5^ Laboratorio de Análisis Espacial de Zonas Costeras, Unidad Multidisciplinaria de Docencia e Investigación-Sisal, Facultad de Ciencias, Universidad Nacional Autónoma de México, Mérida, Yucatán, México

**Keywords:** sea urchins, parrotfish, reef accretion, bioerosion, thermal variability, carbonate budget

## Abstract

Coral reefs deliver vital services via a complex three-dimensional framework sustained by the balance between calcium carbonate production and erosion, or the net carbonate budget state. In many tropical western Atlantic reefs, ecological decline has reduced carbonate production, yielding near-neutral or negative budgets. Yet some reefs retain high coral cover and, theoretically, should also have high net positive budgets, yet often show modest carbonate accumulation. We used the remote reef of Cayo Arenas in the Campeche Bank, Gulf of Mexico, to test whether in reefs under suboptimal (variable) environmental conditions, high coral production is offset by robust bioeroder communities, producing neutral budgets. At 14 sites, we quantified carbonate producers and bioeroders to estimate gross production, bioerosion and net budget states. Despite relatively high live coral cover, mean net carbonate budgets did not differ significantly from a neutral (zero) state, despite a positive net budget (0.7 ± 0.5 SE kg CaCO_3_ m^−2^ yr^−1^) and substantial site-to-site variability (range: −2.3 to 4.3 kg CaCO_3_ m^−2^ yr^−1^). Crucially, this neutrality arose not from depressed biological activity (as in degraded reefs) but from an active equilibrium: elevated carbonate production coupled with substantial bioerosion. These reefs, therefore, represent a contemporary, functional reef state in net stasis. Distinguishing active-neutral reefs, where both production and bioerosion are elevated, from impoverishment-neutral reefs, where both are suppressed, is critical for predicting reef trajectories under environmental change and for targeting management, although near-stasis emerging from high carbonate turnover can appear functionally intact yet operate with limited buffering capacity against net carbonate loss.

## 1. Introduction

Biodiversity and ecosystem services associated with coral reefs largely depend on the reef structural framework, which is formed through the net biogenic accumulation of calcium carbonate (CaCO_3_) over ecological, geomorphic and geological timescales from years to millennia [1]. Reef formation and persistence is underpinned by two fundamental processes: the production and erosion of calcareous structures. The production of CaCO_3_ (gross production) provides the carbonate that builds and cements the reef framework, with scleractinian corals being the primary calcifiers [2]. By contrast, erosional processes remove this material from the reef matrix. Parrotfish and sea urchins are often the primary source of biological erosion (or bioerosion) [3], while physical disturbances can also strip large volumes of carbonate framework off the reef and/or drive reef-building. Resultant rates of gross production and bioerosion contribute to the amount of CaCO_3_ that remains in the reef structure, and the balance is often described as the calcium carbonate net budget or net production [4,5]. In this sense, carbonate budgets have become a tool for quantitatively estimating how gross production and bioerosion processes determine aspects such as habitat provision, coastal protection and conservation status [6].

There are three possible carbonate-dynamic scenarios on reefs [1,7,8]. A positive net budget is characterized by high cover of reef-building corals whose CaCO_3_ accumulation supports framework construction and net reef accretion (scenario I, figure 1) and is generally considered optimal for tracking sea-level change and maintaining key reef functions [9,10]. Conversely, a negative net budget arises when erosion exceeds gross production, producing net loss of CaCO_3_ (scenario III, figure 1); these states are typically associated with ecological degradation, low coral cover and dominance by slow‑calcifying species [11,12]. Here, we focus on gross production and *in situ* bioerosion as the primary drivers of net budget state; physical import and export of sediment can further modify net accumulation independently of these biological rates [7] and are not directly quantified in this study. Between these extremes is reef stasis, where gross production and erosion are balanced and framework accumulation is very slow or negligible. Importantly, this neutral condition can occur at distinct operating points: when both gross production and bioerosion are low (impoverished neutrality, scenario II, figure 1) or when both are high (active neutrality, scenario IV, figure 1). Impoverished neutrality reflects a functionally depleted, degraded system, whereas active neutrality denotes a balanced, structurally robust and functionally active reef. In comparison with impoverished neutrality, active neutrality is driven by high cover of key reef-building corals and a considerable abundance of high-rate bioeroding taxa such as parrotfish and sea urchins [13,14].

**Figure 1.**
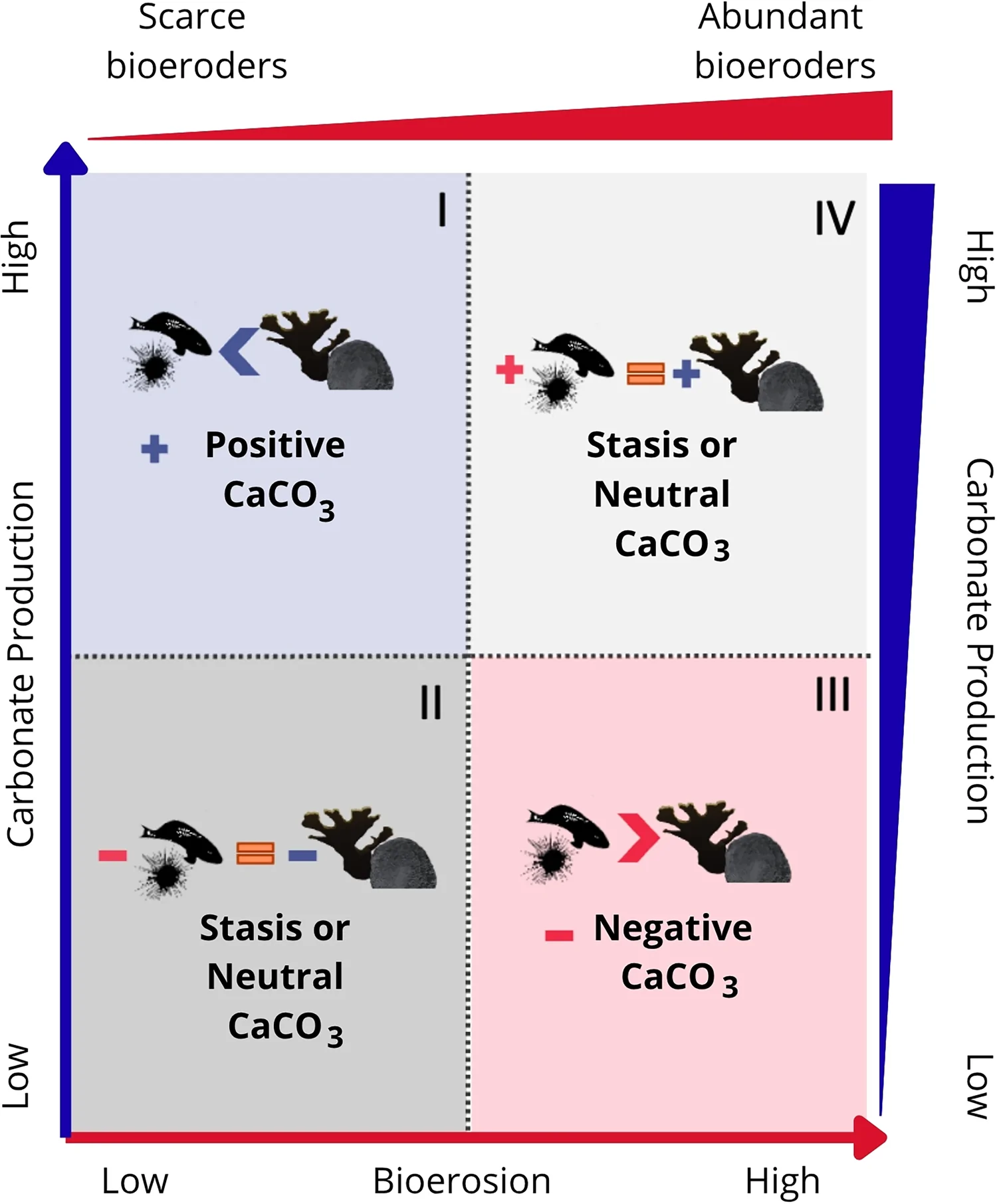
Conceptual model of four possible scenarios for net calcium carbonate budgets, taking into account the ecological context. The right margin shows carbonate production increasing from bottom to top. The top margin indicates the presence or absence of bioeroders, with abundance increasing from left to right. Quadrant I exemplifies a positive, high-budget state, resulting from high gross production by high-functional corals, with bioerosion lower than gross production (bioerosion less than gross production). Quadrant II shows a neutral net budget or impoverished neutrality; it is associated with a context of ecological degradation, where there is little contribution of CaCO_3_ from low-functional corals with CCA, and a low abundance of bioeroders (bioerosion = gross production). Quadrant III refers to a negative net budget caused by an increase in bioeroder abundance that outweighs the low gross production of corals with low reef functionality (bioerosion more than gross production). Quadrant IV refers to an active neutrality state; associated with high ecological activity, where gross production by high-functionality corals is offset by intense bioerosion driven by a high abundance of bioeroders (bioerosion = gross production). Cayo Arenas (this study) sits close to the boundary between quadrants II and IV rather than squarely within either: gross production and bioerosion are both elevated relative to impoverished-neutral reefs, but production remains below the optimal range reported for branching-dominated reefs (see §4). This framework considers gross production and *in situ* bioerosion only; physical erosion and sediment export [7] are not represented and could independently modify net accumulation.

Many tropical western Atlantic reefs currently occupy impoverished‑neutral states (scenario II, figure 1), following acute or chronic coral mortality from bleaching and disease, which have greatly reduced calcifier abundance [15–18]. Concurrent reductions in bioerosion—notably declines in sea urchin and parrotfish densities—have allowed reefs with low gross production to persist in stasis or slight accretion, a pattern common across the region [9,19]. By contrast, active neutrality (scenario IV, figure 1) requires both gross production and bioerosion to remain elevated relative to this degraded baseline and is less examined despite being ecologically plausible, particularly in reefs developed under suboptimal conditions (e.g. turbid, eutrophic or thermally variable environments), which may characterize most of the world's coral reefs [20]. Assemblages in such settings are often dominated by massive and submassive, stress-tolerant corals (e.g. *Orbicella* spp., *Montastraea cavernosa*, *Pseudodiploria* spp.) that can sustain moderate carbonate production despite rarely attaining the high gross production of fast-growing branching taxa [21,22]. Moreover, suboptimal conditions often reduce species-level skeletal extension and density relative to optimal conditions, further limiting community calcification [23–25]. Under these conditions, production that is elevated relative to impoverished-neutral reefs, yet still structurally constrained relative to production on optimal, branching-dominated reefs, may be effectively offset by healthy and more active rates of bioerosion [26,27], resulting in near-neutral carbonate budgets maintained through high process turnover rather than through functional collapse. This highlights the necessity of using local, contemporary measurements of gross production and bioerosion to accurately characterize reef carbonate dynamics.

To evaluate the operability of active neutrality (scenario IV, figure 1), we quantified gross production and bioerosion at Cayo Arenas, in the southern Gulf of Mexico. This small, relatively isolated reef system remains in good ecological condition, with high abundances of key reef-building corals and a healthy population of bioeroders (figure 2) [13,29]. Nevertheless, Cayo Arenas lies in a region with variable environmental conditions, most notably elevated intra-annual variability in sea surface temperature, which reduces coral calcification [30–32]. We therefore hypothesize that Cayo Arenas exhibits active, rather than impoverished, neutrality driven by the co-occurrence of robust carbonate producers and active bioerosion under variable environmental conditions. To test this hypothesis, we conducted ecological, gross production and bioerosion process characterizations of sites across this system with the *ReefBudget* methodology [33], incorporating locally measured calcification rates for *Orbicella* spp. and extensive sea urchin surveys to account for this cryptic, nocturnal bioeroder [13]. We then place our results in the context of comprehensive Western Caribbean data to contextualize and discuss the implications of our findings.

**Figure 2.**
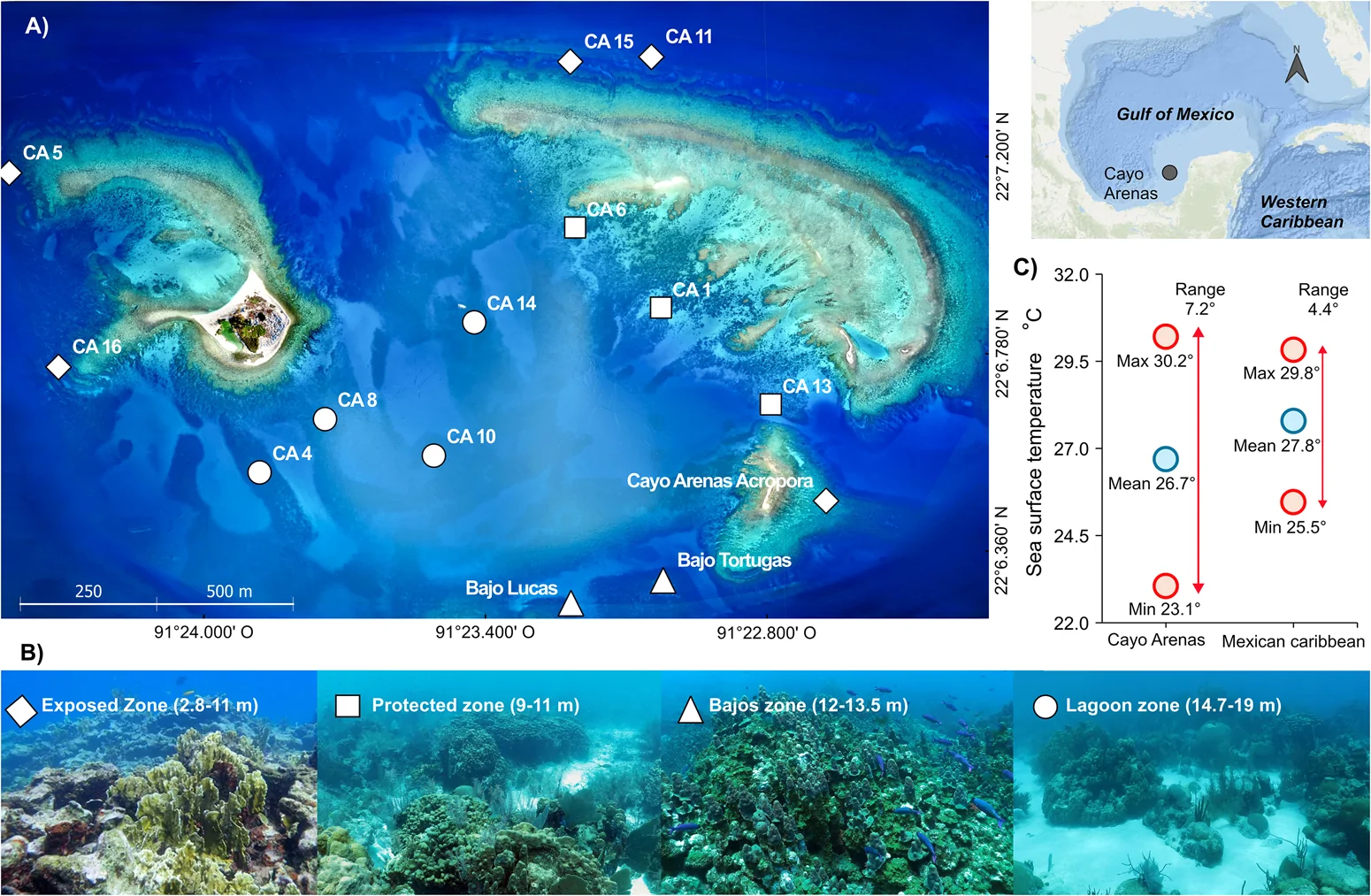
Location of Cayo Arenas and general information about the reef. Distribution of the 14 sampled sites (A) and their classification into four reef zones (B). Also shown are the maximum, minimum and average sea temperatures, as well as the range (°C) for Cayo Arenas and the Mexican Caribbean for comparison (C). The top-right inset shows the positioning of Cayo Arenas in the Gulf of Mexico. The sea temperature data were obtained from Bio-ORACLE satellite data [28]. The photo of Cayo Arenas is an orthomosaic created from drone imagery. The drone flights and orthomosaic processing were conducted by Lorenzo Alvarez-Filip.

## 2. Material and Methods

### 2.1. Study area

Cayo Arenas is a remote platform reef approximately 200 km from the Yucatán coast, has an area of 10 km^2^ [29], and forms part of the Campeche Bank reef system in the southeast of the Gulf of Mexico (figure 2). It comprises a complex of reef structures that reach depths of up to 30 m and remain interconnected with one another [34]. The underlying platform of the Campeche Bank is composed mainly of solidified carbonate materials [35] and exists in a region with variable environmental conditions, including large intra-annual variability in sea surface temperature (7°C range), nutrient concentrations and other environmental parameters (figure 2) [28]. These result in low coral calcification and probably limit vertical reef growth [36,37]. The northeast structure of Cayo Arenas is the largest formation and has two emergent areas; the southeast structure is the smallest and has a small emerging area. These two structures receive the greatest impact from wind and wave energy (figure 2). The eastern structure, which has a lagoon on the windward side and a cay with vegetation, is in the most protected part of the system [35]. Cayo Arenas reefs are dominated by a high abundance of large scleractinian massive corals, including *O. faveolata, O. annularis*, *Montastraea cavernosa*, *Colpophyllia natans* and *Siderastrea* spp. [10,38].

### 2.2. Data collection

For this study, 14 sites distributed across the four reef zones of Cayo Arenas (exposed, protected, bajos and lagoon; figure 2) were surveyed in four expeditions between July 2017 and August 2024. Some sites (CA4, CA13, Bajo Tortugas and CA1) were monitored for more than 1 year; however, because we did not examine temporal changes, we used only the most recent year with information for both producers and bioeroders. The four reef zones were defined as follows. The exposed zone occupies the fore-reef, where wind, waves and currents generate relatively high turbulence; its shallow platform reaches a maximum depth of 5.4 m and has a gentle slope that supports species typical of shallow, turbulent water, such as *Acropora palmata* and *Millepora complanata*, beyond which the slope steepens and hydrodynamic energy gradually decreases with depth. The protected zone extends from approximately 5–9 m depth and is characterized by very low hydrodynamic energy that limits the development of branching corals, with coral patches occurring over a sandy substrate and connecting directly to the deeper protected area. The bajos zone comprises submerged reefs not sheltered by reef crests; its environmental conditions and coral diversity resemble those of the deep protected zone, but coral cover forms extensive carpets of massive corals rather than isolated patches. The lagoon zone, identified specifically at Cayo Arenas, reaches depths of up to 20 m and has a sandy substrate with patches of massive corals and slightly greater hydrodynamic energy than the protected zone. Because reef geomorphology, current intensity and depth varied substantially across sites, complicating standardized monitoring, the number of transects per group of organisms also varied across sites, prioritizing broader spatial coverage over uniform replication within each site (see the supplementary material, tables S1 and S2) [39].

At each site, a census was conducted using the ReefBudget v2 method for the Caribbean Sea [33] in which the line-intercept method was applied: the substrate type and length of each coral colony and other benthic component intercepted along the transect line were recorded to the nearest centimetre, including at the deeper site CA10 (supplementary material, table S1). For bioeroders, a belt transect method was used (10 × 1 m) for sea urchins and endolithic sponges, and 30 × 2 m (60 m²) for all non-cryptic reef fish species, with particular focus on parrotfishes given their functional relevance [33]. Because visual belt-transect surveys of endolithic sponges and microborers cannot detect boring activity beneath the substrate surface, this approach probably underestimates their true contribution to total bioerosion [33]; we consider this further in the Discussion. In the case of fish, the species, number of individuals and fork length of each individual were recorded (fork length estimated visually) and classified by size (less than 5, 8, 15 cm and so on in 10 cm increments up to more than 120 cm). In addition to parrotfish, we also recorded the abundance and size of commercially important fish (groupers and snappers) and the main sea urchin predators (see the supplementary material, figure S1, for details).

Furthermore, we conducted night-time monitoring to survey sea urchins at four sites (Bajo Tortugas, CA1, CA14 and CA15), as bioerosion rates by this group, based on daytime surveys, typically underestimate their abundance owing to their diurnal cryptic lifestyle [13,40]. Each surveyed site represented one of the four reef zones (figure 2). To make data collection with diving torches more efficient, the transect dimensions were modified to 20 × 0.5 m, while maintaining the sampling area (10 m^2^) [13]. Surveys started at least 90 minutes after sunset. For logistical and safety reasons, it was not possible to survey all sites at night.

### 2.3. Ecological characterization of Cayo Arenas

Although the primary focus of this study is on reef carbonate budgets, we conducted a broader ecological characterization of Cayo Arenas, based on the same visual census data used throughout the study. We included metrics of live coral cover, the total absolute cover (% ± SE) of five coral groups (massive, submassive, branching, digitate and other [33,41]; supplementary material, figure S2A), the density (ind m^−2^ ± SE) and/or biomass (kg 100 m^−2^ ± SE) of bioeroders, and the biomass of commercially important fishes as well as sea urchin predators (supplementary material, figure S3A). These metrics were used solely to provide ecological context for the carbonate budget, rather than as a stand-alone assessment of reef status. Fish biomass was estimated from species size and abundance using allometric equations and parameters from Froese & Pauly [42]. We estimated the population density of sea urchins (*Diadema*, *Echinometra* and *Eucidaris*) with data from night surveys.

We used the Mexican Caribbean as a reference to situate our findings in a broader ecological context, given that it is well-studied, geographically closest and most connected to the Campeche Bank [43,44]. For fish biomass, Mexican Caribbean sites were further classified as ‘Well-Managed’ or ‘Under-Managed Fisheries’ following Espinosa-Andrade *et al.* [45], using mean values from the 2017–2021 literature (full site list and classification: supplementary material, table S4). Since sea urchins are not an important species in Caribbean fisheries, we do not distinguish between these areas and group all sites as the Mexican Caribbean.

### 2.4. Reef carbonate budget estimation

We estimated carbonate budgets by quantifying gross production and bioerosion using the *ReefBudget* v2 protocol [33]. Net carbonate budget was expressed as the sum of all production minus total bioerosion, standardized to kg CaCO_3_ m^−2^ yr^–1^. Gross production was estimated primarily from field-derived benthic cover (including CCA) using line‑intercept transects, combined with species-specific skeletal density and linear growth extension rates from the *ReefBudget* v2 database for the Gulf of Mexico and Caribbean [33].

To better reflect local conditions, we adjusted density and linear-growth values from the *ReefBudget* database via a geographic filter (Gulf of Mexico → Campeche Bank → Cayo Arenas; supplementary material, table S3). This provided site- and region-specific information for *Orbicella* spp. (*O. annularis* for Cayo Arenas; *O. faveolata* for the Gulf of Mexico). For other morphotypes (massive, submassive, digitate, branching), extension rates were taken from Gulf of Mexico data, and skeletal density from Caribbean regional averages (supplementary material, table S3).

Because light attenuation affects calcification [46], we incorporated depth (light availability) effects on the dominant reef-builder *O. faveolata* using the Gutiérrez-Estrada *et al.* [24] model, which predicts calcification from the PAR attenuation coefficient (K_dPAR_). We used K_dPAR_ = 0.077 m^−1^ for Cayo Arenas [28] and mean sampling depths (supplementary material, table S1) to estimate depth‑adjusted calcification rates for *O. faveolata* at each site, constraining modelled calcification not to exceed observed averages for 5–10 m depths (0.85 g cm^−2^ yr^−1^) [32]. These site‑specific *O. faveolata* calcification coefficients were incorporated into the *ReefBudget* gross‑production formulas. We applied depth adjustments only to *O. faveolata* because species‑specific calcification–light relationships are not available for other taxa.

Bioerosion rates followed *ReefBudget* v2 parameters and equations [33]. Parrotfish erosion was estimated from bite rate (bites day^−1^), fish size and life stage, bite volume (cm^3^), proportion of bites that remove substrate and substrate density (g cm^−3^), yielding kg CaCO_3_ day^−1^, which was standardized to area and year. Endolithic sponge erosion used species‑specific substrate area (m^2^) and published annual bioerosion rates [47], divided by transect area to give kg CaCO_3_ m^−2^ yr^−1^. Microbioerosion used the proportion of hard substrate available for microboring (excluding live coral and algal mats) with published average annual rates (kg CaCO_3_ m^−2^ yr^−1^) [48–50]. Sea‑urchin erosion was estimated at species level and test‑size distributions on transects, using published regressions relating test size to erosion (g urchin^−1^ day^−1^) [33], and converted to kg CaCO_3_ m^−2^ yr^−1^.

For sites where sea urchins were recorded only during daytime surveys, we applied day-night conversion factors derived from one site from each of the four reef zones where both diurnal and nocturnal surveys were conducted (see the supplementary material, table S1) [13]. At sites with their own nocturnal survey (Bajo Tortugas, CA1, CA14), the site-level mean nocturnal rate was applied to every daytime transect at that site, as daytime and nocturnal surveys did not share the same transect layout. These zone‑specific conversion factors were used, except for Bajo Lucas, for which we applied nocturnal rates from the neighbouring Bajo Tortugas, as subsequent surveys showed that both reefs had very similar urchin communities in terms of density and composition. Total bioerosion per transect was the sum of all group contributions; only nocturnal sea‑urchin estimates were used in the reported total bioerosion, whereas daytime rates were retained for other bioeroder groups.

We tested the influence of (i) using reduced, area‑specific *O. faveolata* gross‑production rates and (ii) including diurnal versus nocturnal sea‑urchin bioerosion across reef zones. Because the data violated the assumptions of normality and homoscedasticity, we used paired Wilcoxon signed‑rank tests. For *O. faveolata*, we compared gross production (kg CaCO_3_ m^−2^ yr^−1^) from Gulf of Mexico literature values (supplementary material, table S3) with calcification rates estimated from Gutiérrez-Estrada *et al.* [24]; and for sea‑urchin erosion, daytime and night-time rates were compared. To formally evaluate whether the reef-wide net carbonate budget differed from a strictly neutral (zero) state, we tested site-level mean net budgets against zero using a one-sample Wilcoxon signed-rank test. All analyses were performed in R v. 4.3.1 [51].

## 3. Results

### 3.1. Cayo Arenas is a reef in good ecological condition

Cayo Arenas has a high average (±SE) coral cover of 29.5 ± 1.4% (figure 3A), with the highest values at the bajos (34.8 ± 2.5%) and protected zones (35.9 ± 3.4%) and the lowest at the lagoon (29.3 ± 2.4%) and exposed zones (23.7 ± 2.2%). Overall, the coral assemblage is dominated by massive taxa (19.6 ± 1.5%), most notably *Orbicella* spp. (13.7 ± 1.3%), particularly in zones protected from wave energy. By contrast, branching coral cover, associated with the highest calcification rates in the region, is low (0.78 ± 0.33%) in this system and is represented exclusively by *Acropora palmata*, which was restricted to two sites in the exposed zone. Non-reef-building coral species had the lowest cover (1.0 ± 0.3%). CCA cover was consistently high across reef zones (12.5 ± 1.4%), with peak values in the bajos (15.8 ± 2.3%) and exposed (20.4 ± 3.0%) zones.

**Figure 3.**
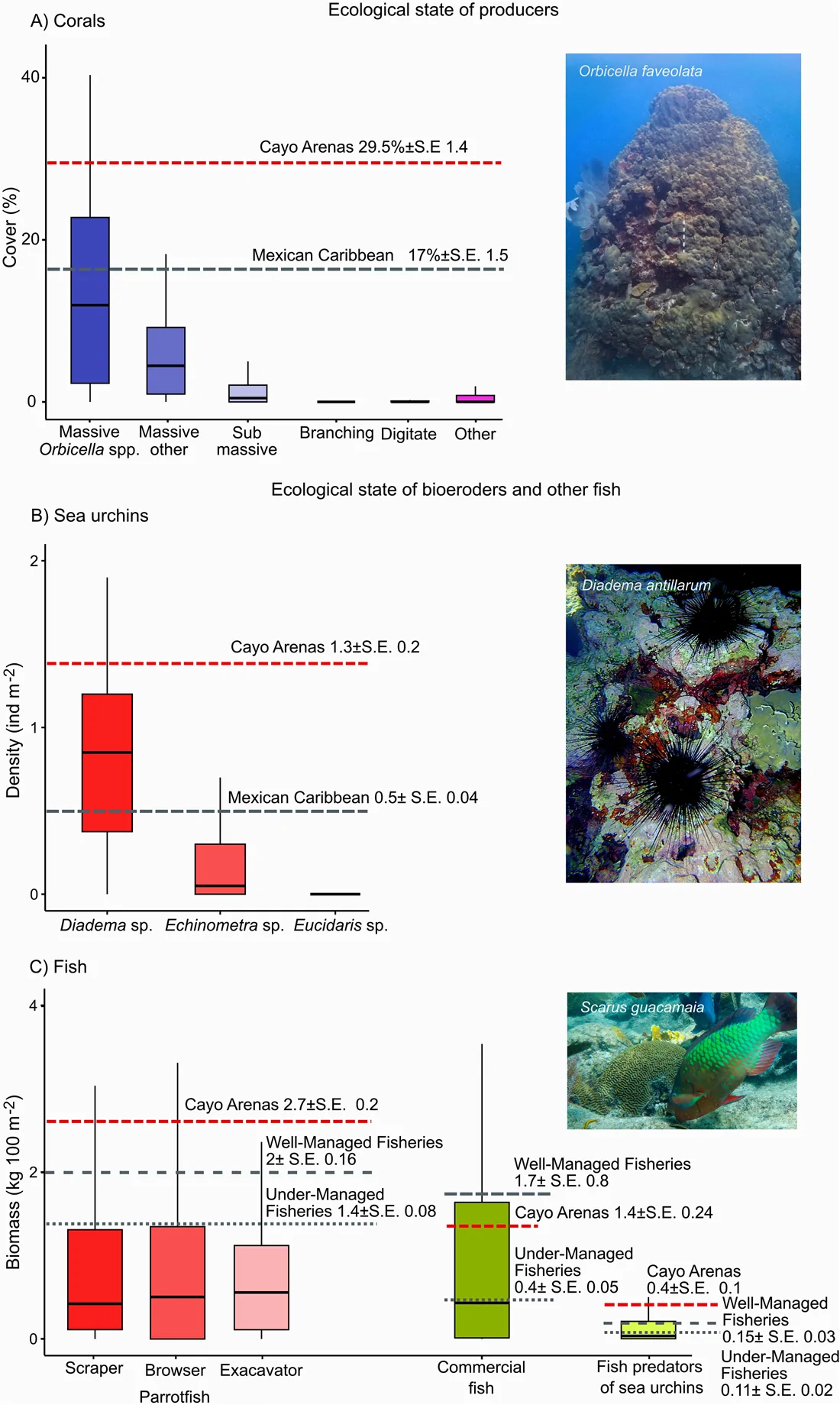
Ecological characterization of Cayo Arenas reef. Comparisons were made with the Mexican Caribbean for coral cover and populations of eroding urchins and fish. The red dashed lines indicate the averages for Cayo Arenas. The grey dashed lines indicate the average for the Mexican Caribbean for coral cover and urchins, whereas for fish, they indicate the Well-Managed Fisheries in the Mexican Caribbean. The grey dotted lines indicate the Under-Management Fisheries in the Mexican Caribbean. The percentage of coral cover in the Mexican Caribbean was taken from Molina-Hernández *et al.* [19]. Box plots indicate the interquartile range that contains 50% of the data. Lines within the boxes indicate the median. The lower and upper whiskers extend 1.5 of the interquartile range and show the variability of the data. A striped PVC pole (5 cm per band; 45 cm total length) is shown against the *O. faveolata* colony for scale. Photographs by Lorenzo Alvarez-Filip.

The average biomass of parrotfish (family Scaridae, mainly *Sparisoma* and *Scarus* spp.) in Cayo Arenas was 2.7 ± 0.2 kg 100 m^−2^ (figure 3C). The zone with the highest biomass was the exposed zone (4.0 ± 0.5 kg 100 m^−2^) and the zone with the lowest biomass was the bajos zone (1.3 ± 0.3 kg 100 m^−2^). The group of parrotfish with the highest biomass was the scraper, with 0.95 ± 0.11 kg 100 m^−2^, followed by the excavator, represented solely by the species *Sparisoma viride*, with 0.9 ± 0.1 kg 100 m^−2^ and the browser, with 0.85 ± 0.10 kg 100 m^−2^. The average biomass of parrotfishes in Cayo Arenas was higher than that observed in the Caribbean, exceeding that of Under-Managed sites by approximately 50% and Well-Managed sites by approximately 25% (figure 3C).

The average biomass of commercially important fishes was 1.40 ± 0.24 kg 100 m^−2^ (figure 3C), highest in the protected zone (2.1 ± 0.9 kg 100 m^−2^) and lowest in the bajos zone (1.1 ± 0.5 kg 100 m^−2^). Sea urchin predator fishes averaged 0.40 ± 0.01 kg 100 m^−2^ (figure 3C), highest in the exposed zone (0.7 ± 0.3 kg 100 m^−2^) and lowest in the protected zone (0.2 ± 0.1 kg 100 m^−2^). Relative to reference values from the Mexican Caribbean, parrotfish and sea urchin predator biomass at Cayo Arenas exceeded both Well-Managed and Under-Managed Fisheries sites, while commercially important fish biomass was comparable to (slightly below) Well-Managed sites and well above Under-Managed sites (figure 3C; supplementary material, table S4), consistent with strong top-down control over the sea urchin population.

In the case of eroding sea urchins, Cayo Arenas recorded a population density of 1.3 ± 0.2 ind m^−2^ (figure 3B). The zone with the highest sea urchin density was the bajos (2.80 ± 0.95 ind m^−2^), whereas the zone with the lowest density was the lagoon (0.02 ± 0.02 ind m^−2^). *Diadema antillarum* had the highest population density (1.0 ± 0.2 ind m^−2^), followed by the *Echinometra viridis* (0.2 ± 0.6 ind m^−2^), *E. lucunter* (0.01 ± 0.24 ind m^−2^) and finally *Eucidaris tribuloides* (0.02 ± 0.05 ind m^−2^). *Diadema antillarum* was also highly associated with the bajos zone, where it reached its highest density (2.5 ± 0.9 ind m^−2^).

### 3.2. Production and bioerosion rates are in relative equilibrium

Cayo Arenas gross production rate averages 3.5 ± 0.3 kg CaCO_3_ m^−2^ yr^−1^, which is attributed almost entirely to the contribution of corals, with CCA accounting for only approximately 1% (0.05 ± 0.01 kg CaCO_3_ m^−2^ yr^−1^). The species with the highest contribution to gross production is *O. faveolata* (1.0 ± 0.1 kg CaCO_3_ m^−2^ yr^−1^; supplementary material, figure S2B). At the same time, the mean bioerosion rate for the system was only slightly lower than the gross production (2.4 ± 0.2 kg CaCO_3_ m^−2^ yr^−1^). Parrotfish made the greatest contribution to net bioerosion (1.5 ± 0.2 kg CaCO_3_ m^−2^ yr^−1^), primarily driven by *Sp. viride* and *Scarus vetula* in the exposed and protected zones (supplementary material, tables S2 and S5). Sea urchins also exhibited a comparably high bioerosion rate (1.0 ± 0.2 kg CaCO_3_ m^−2^ yr^−1^), particularly by *D. antillarum* in reefs with higher coral complexity in the bajos zone (supplementary material, figure S5 and tables S6 and S7). The contributions of endolithic sponges (0.08 ± 0.08 kg CaCO_3_ m^−2^ yr^−1^) and microbioerosion (0.1 ± 0.0 kg CaCO_3_ m^−2^ yr^−1^) to the overall bioerosion were modest (supplementary material, figure S3C and tables S2, S9 and S10), though this probably underestimates the true contribution of endolithic bioerosion given the limitations of visual belt-transect surveys for this group (see Material and Methods).

Overall, the net budget was close to stasis (0.7 ± 0.5 kg CaCO_3_ m^−2^ yr^−1^; range: −2.3 to 4.3; *n* = 14 sites) and did not differ significantly from zero (one-sample Wilcoxon signed-rank test, *V* = 32, *p* = 0.22), confirming a statistically neutral state despite the positive point estimate, as moderate bioerosion rates closely counterbalanced gross CaCO_3_ production. The zones with the highest net budgets were the protected and lagoon zones (3.1 ± 0.7 and 1.2 ± 0.8 kg CaCO_3_ m^−2^ yr^−1^, respectively), where the processes of bioerosion seemed to be less active (supplementary material, figures S4 and S5 and table S2), while the bajos and exposed zones had the lowest net budgets (0.9 ± 0.7 and −1.1 ± 0.5 kg CaCO_3_ m^−2^ yr^−1^, respectively), largely owing to the high level of parrotfish and sea urchin bioerosion observed on those sites (supplementary material, figures S4 and S5 and table S2). Although there is high variability at the transect level (figure 4; supplementary material, figure S4), 90% of sites have a relatively low net budget state (ranging from −2.5 to 2.5 kg CaCO_3_ m^−2^ yr^−1^; supplementary material, table S2).

**Figure 4.**
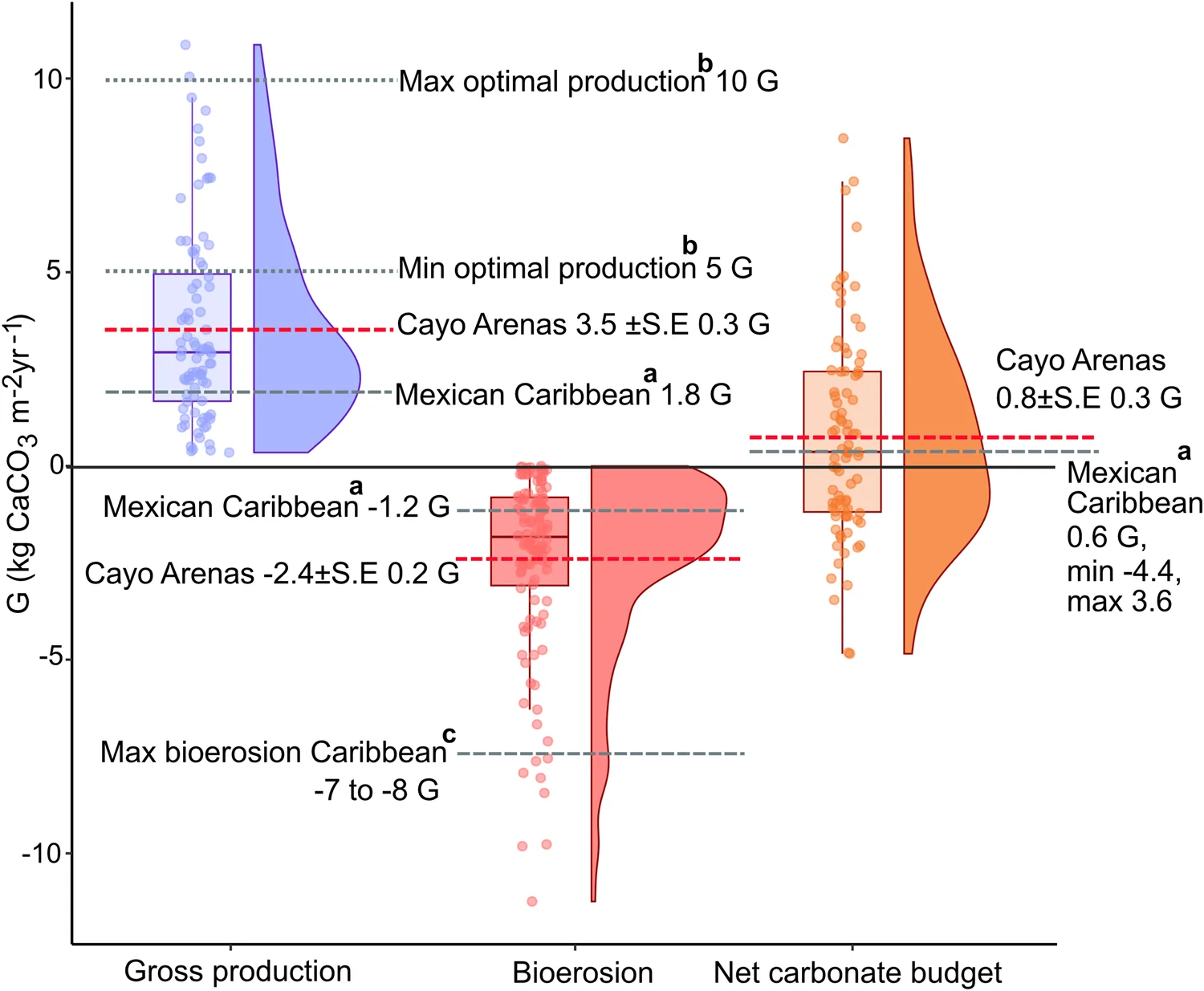
Gross production rates, bioerosion and carbonate net budget of Cayo Arenas. Averages for Cayo Arenas are shown (dashed red lines). Points are transect-level values. References were included to compare the Mexican Caribbean (Molina-Hernández *et al.* [19]^a^; dashed grey lines), the historical maximum bioerosion estimate for the Caribbean (Perry *et al.* [52]^c^; dashed grey lines), and optimal gross production (5–10 kg CaCO_3_ m^−2^ yr^−1^ = G) estimated and considered for tropical forereefs (Vecsei [53]^b^; dotted grey lines). Box plots indicate the interquartile range, which contains 50% of the data. Lines within the boxes indicate the median. The lower and upper whiskers extend to 1.5 times the interquartile range, indicating variability in the data. Distribution diagrams are shown above the box plots; each point represents the value for a given transect. Violin plots show probability density.

## 4. Discussion

Our findings confirm that scenarios with neutral net budgets, characterized by net carbonate production values close to zero, do not necessarily indicate degraded habitats with low coral cover and depleted fish stocks (scenario II, figure 1). Instead, we found that Cayo Arenas functions as a robust reef system (*sensu* [54]), displaying high cover of key reef-building corals and the presence of herbivores (e.g. parrotfish and *D. antillarum*) along with high biomass levels of high-trophic-level species (e.g. groupers, snappers) comparable to those in Well-Managed Fisheries (figure 3C). Here, production stasis appears driven not by ecological collapse but by relatively low calcification rates of dominant coral species—probably a consequence of suboptimal environmental conditions, rather than by reduced coral abundance (figure 1) [32,55]. Concurrently, high abundances of parrotfish and sea urchins indicate strong potential for removal of framework carbonate and sediment; the elevated sea urchin signal is particularly apparent at night, when individuals shelter by day in complex reef structure (supplementary material, figure S5 and tables S2 and S6) [13]. Thus, in functionally robust reefs such as Cayo Arenas, ecological interactions can sustain a net budget that is statistically indistinguishable from neutral, despite wide site-to-site variability (−2.3 to 4.3 kg CaCO_3_ m^−2^ yr^−1^), driven by the coexistence of elevated carbonate production and active bioerosion, rather than by loss of ecological functionality.

Cayo Arenas is better classified as a marginal reef [21]: although its recorded sea-surface temperature range (23.1–30.2°C, mean 26.7°C) remains within the broad thresholds generally considered suitable for reef growth (18–31.5°C) [56], the approximately 7°C intra-annual range represents comparatively large and recurrent thermal variability for a shallow reef system (figure 2). Such variability exposes corals to recurrent stress yet fosters assemblages dominated by stress‑tolerant taxa capable of maintaining structural integrity, relatively high coral cover (more than 30%) and geo‑ecological functions. These massive species typically have lower calcification rates than fast‑growing acroporids found in more ‘optimal’ settings [57], but their morphology and biological traits (thick tissue and reduced skeletal density) confer resilience to environmental fluctuations [58]. Stress tolerance in extreme or marginal settings is not exclusive to massive morphologies; however, branching species can also show elevated tolerance relative to conspecifics on benign reefs [59]. The *Orbicella* complex, a historical reef builder throughout the Holocene in the western Atlantic [60], contributes substantially to modern reef CaCO_3_ production [24,41], highlighting its ecological importance and adaptive capacity. High *Orbicella* cover may reflect allocation of calcification to extended skeleton growth despite porous skeletons [30,31,55]. Furthermore, colony‑level plasticity in physiology and morphology enables these species to persist under variable light, depth, temperature and turbidity [30,61], with direct consequences for community composition and contribution to reef carbonate construction.

Gross carbonate production at Cayo Arenas was below the reported optimal regional range of 5–10 kg CaCO_3_ m^−2^ yr^−1^ [53], despite exhibiting high cover of reef-building coral species. This reflects the dominance of *Orbicella faveolata*, a massive reef-building coral whose calcification rates are lower than those of fast-growing branching taxa, particularly under seasonally temperature-stressed conditions. The low gross production values also reflect the use of the calcification model of *O. faveolata* to estimate its production (see Material and Methods; supplementary material, figure S5); because this species is dominant, this consequently reduced the estimated gross production of the study area. Low gross production under suboptimal conditions is more consistent with a marginal reef classification [21] than with the high-production, high-diversity extreme-reef endpoint of that same framework. At greater depths and under turbid conditions, calcification decreases as light availability does [24,62,63]. Long-term exposure to temperature variability may increase thermal tolerance in corals at Cayo Arenas relative to those in the Mexican Caribbean [64,65]. Independently of any such thermal effects, however, this reef's persistence remains a concern because the coral species that dominate Cayo Arenas are highly susceptible to diseases that have caused significant mortality in the Caribbean Sea [15,16,66].

A notable result of this study is that sea urchins contributed nearly half of the total bioerosion at Cayo Arenas (*D. antillarum* density is more than double the Mexican Caribbean average; figure 3B), a contrast to many Caribbean reefs still recovering from the 1980s *Diadema* die‑off [67], and consistent with reports of robust sea urchin populations in other reefs of the Gulf of Mexico (e.g. Veracruz, Banco Stetson, Flower Gardens) [68–70]. The sea urchin bioerosion rates throughout Cayo Arenas were significantly higher during night-time surveys although their populations were also quite abundant during the daytime (supplementary material, figure S5B) [13]. While high local bioerosion might signal an imbalance driven by excessive sea urchin density, often linked to overfishing and loss of predators [71], this appears unlikely here. The fact that fish biomass at Cayo Arenas is comparable to that of Well-Managed Fisheries in the Caribbean (figure 3C) and the availability of structurally complex refuges [13] suggest a currently stable and controlled urchin population. Nonetheless, given the high erosion rates in some areas of Cayo Arenas and elsewhere in the Gulf of Mexico, intensified fishing, which removes top-down control, could rapidly shift the system towards highly erosive states observed elsewhere.

Parrotfishes were the primary bioeroders at Cayo Arenas, consistent with their well-documented role as the dominant bioeroding group in Atlantic and other reef systems (e.g. [72]). Among the most abundant species were *Sp. viride* and *Sc. vetula*, which, in addition to being the two species with the highest eroding potential in the Atlantic Ocean, are also the main sediment producers [3]. This is important because these species are currently highly vulnerable to fishing, which could compromise the reef's sediment supply [3]. Parrotfish bioerosion at Cayo Arenas fell within values reported for the Campeche Bank (approx. 0.5–4 kg CaCO_3_ m^−2^ yr^−1^) [14] but exceeded rates reported for nearshore reefs (Mexican Caribbean, Veracruz; 0.4–0.7 kg CaCO_3_ m^−2^ yr^−1^) [10,19]. By contrast, microbioerosion and endolithic sponge erosion made only minor contributions to the budget, although their rates were within ranges reported elsewhere [10]. This differs from some studies in which endolithic sponges play larger roles, often when sea urchins are absent, underscoring context‑dependent shifts among bioeroder groups [73,74].

Overall, Cayo Arenas exhibits a net carbonate budget that is statistically indistinguishable from neutral. This balance is driven by a coral community dominated by high-cover, moderately calcifying massive taxa, particularly *Orbicella* spp., rather than fast-growing branching corals, a composition consistent with the comparatively low coral diversity of this system. Locally reduced per-colony calcification under suboptimal environmental conditions further constrains carbonate production, whereas healthy populations of parrotfishes and sea urchins sustain relatively high bioerosion rates (figures 3 and 4; supplementary material, figure S5 and table S2). Consequently, Cayo Arenas lies near the boundary between active and impoverished neutrality (figure 1), rather than fitting squarely within either category: carbonate production exceeds that of degraded, impoverished-neutral reefs but remains lower than in branching-coral-dominated systems representing the active-neutral endpoint. This differs from impoverished neutrality at many Caribbean sites, where neutrality results from coral-community deterioration [2,10] and reduced bioerosion rates [19,52]. Remote reefs with strongly positive budgets typically report much higher production rates that outpace erosion [2,12,75,76], but those estimates often rely on regional averages and do not account for depth‑related or site‑specific reductions in calcification. Notably, despite Cayo Arenas having considerably higher live coral cover than the regional average (29.5 ± 1.4% vs. 17.0 ± 1.5% in the Mexican Caribbean; figure 3A), its mean net budget (0.7 ± 0.5 kg CaCO_3_ m^−2^ yr^−1^) is closely comparable to that reported for the Mexican Caribbean (0.6 kg CaCO_3_ m^−2^ yr^−1^; figure 4), reinforcing that elevated coral cover does not by itself translate into a proportionally elevated carbonate budget once concurrent bioerosion is taken into account. In our study, adjusting *O. faveolata* calcification to local conditions and including nocturnal urchin bioerosion lowered net estimates. This illustrates how local, contemporary measurements of calcification and bioerosion are essential for accurate carbonate‑budget assessments and for predicting reef responses to increasing environmental threats. We therefore emphasize the importance of continuing to generate local information on calcification and bioerosion and including it in the estimation of carbonate net budgets, as this will allow us to gain more accurate insights into reefs' adaptive responses to growing threats, such as global warming. We note that our budget estimates are bounded by the processes we were able to measure. Physical erosion and sediment export [7] were not quantified and could further modify net accumulation independently of the gross production and *in situ* bioerosion rates reported here, and our visual survey-based estimates of endolithic bioerosion probably underestimate its true contribution (see Material and Methods). Future work incorporating these unmeasured loss and transport terms would refine the carbonate budget presented here.

There are two relevant implications to consider for sites, such as Cayo Arenas, that have relatively good ecological conditions but near-neutral carbonate net budgets. First, it is probable that carbonate net budgets at this site are highly sensitive to drastic changes in some components of the system. For example, if a lethal disease outbreak or a mass bleaching causes widespread coral mortality in susceptible species (e.g. [15,16]), net carbonate budgets could become net erosional. This would result not only from the loss of living tissue and the associated decline in gross carbonate production, but also from the exposure of skeletons of dominant massive corals such as *O. faveolata*, whose skeletal traits render them highly susceptible to intense bioerosion [74,77]. In the presence of robust communities of external bioeroders, such a shift could rapidly drive the system into a strongly net-negative carbonate state, with implications for rapid framework degradation. Second, in the context of rapid anthropogenic climate change, the rates of vertical accretion at Cayo Arenas are nowhere near projected rates of sea-level rise [10]. Consequently, even apparently resilient reefs will lag in long‑term growth and probably will fail to keep pace with sea-level rise [10,78]. This may lead to alterations in reef ecology, particularly in shallow and deep reef assemblages, which are now characterized by reduced gross carbonate production. Preserving the ecological integrity of these remote reefs should therefore be a priority in the coming years, as reefs such as Cayo Arenas remain largely unexplored biological reservoirs. Furthermore, given their healthy populations of key reef-building corals and functional bioeroder communities, they could function as potential refuges for the restoration of reef-building coral populations specifically, even though community-level species diversity at Cayo Arenas remains comparatively low.

## Supplementary Material



## Data Availability

The dataset underlying the analyses is now publicly available [39]. Supplementary material is available online [79].
